# Saturation Mutagenesis and Molecular Modeling: The Impact of Methionine 182 Substitutions on the Stability of β-Lactamase TEM-1

**DOI:** 10.3390/ijms25147691

**Published:** 2024-07-13

**Authors:** Vitaly G. Grigorenko, Alexandra V. Krivitskaya, Maria G. Khrenova, Maya Yu. Rubtsova, Galina V. Presnova, Irina P. Andreeva, Oxana V. Serova, Alexey M. Egorov

**Affiliations:** 1 Chemistry Department, Lomonosov Moscow State University, 119991 Moscow, Russia; grigorenkovg@my.msu.ru (V.G.G.); myr@enz.chem.msu.ru (M.Y.R.); gkovba@enzyme.chem.msu.ru (G.V.P.); imtek1@mail.ru (I.P.A.); aegorov@enz.chem.msu.ru (A.M.E.); 2 Bach Institute of Biochemistry, Federal Research Centre “Fundamentals of Biotechnology” of the Russian Academy of Sciences, 119071 Moscow, Russia; al_krivickaya@mail.ru; 3 Shemyakin-Ovchinnikov Institute of Bioorganic Chemistry of the Russian Academy of Sciences, 117997 Moscow, Russia; oxanaserova@gmail.com

**Keywords:** antibiotic resistance, β-lactamase TEM-1, saturating mutagenesis, M182X mutation, thermostability, molecular modeling

## Abstract

Serine β-lactamase TEM-1 is the first β-lactamase discovered and is still common in Gram-negative pathogens resistant to β-lactam antibiotics. It hydrolyzes penicillins and cephalosporins of early generations. Some of the emerging TEM-1 variants with one or several amino acid substitutions have even broader substrate specificity and resistance to known covalent inhibitors. Key amino acid substitutions affect catalytic properties of the enzyme, and secondary mutations accompany them. The occurrence of the secondary mutation M182T, called a “global suppressor”, has almost doubled over the last decade. Therefore, we performed saturating mutagenesis at position 182 of TEM-1 to determine the influence of this single amino acid substitution on the catalytic properties, thermal stability, and ability for thermoreactivation. Steady-state parameters for penicillin, cephalothin, and ceftazidime are similar for all TEM-1 M182X variants, whereas melting temperature and ability to reactivate after incubation at a higher temperature vary significantly. The effects are multidirectional and depend on the particular amino acid at position 182. The M182E variant of β-lactamase TEM-1 demonstrates the highest residual enzymatic activity, which is 1.5 times higher than for the wild-type enzyme. The 3D structure of the side chain of residue 182 is of particular importance as observed from the comparison of the M182I and M182L variants of TEM-1. Both of these amino acid residues have hydrophobic side chains of similar size, but their residual activity differs by three-fold. Molecular dynamic simulations add a mechanistic explanation for this phenomenon. The important structural element is the V159-R65-E177 triad that exists due to both electrostatic and hydrophobic interactions. Amino acid substitutions that disturb this triad lead to a decrease in the ability of the β-lactamase to be reactivated.

## 1. Introduction

Production of β-lactamase enzymes is the main mechanism of resistance of Gram-negative pathogens against β-lactam antibiotics, which account for approximately 60% of the global market of antibacterial drugs [1,2]. β-Lactamase-catalyzed hydrolysis of the amide bond in the antibiotic’s four-membered β-lactam ring results in the loss of its antimicrobial activity. The first enzyme with activity towards penicillin was discovered in *Escherichia coli* even before it was utilized in clinical medicine [3].

The active use of antibiotics in clinical practice and veterinary medicine contributed to the evolution of β-lactamase-producing bacteria and their widespread distribution [4,5]. The prevalence of antibiotic-resistant pathogenic bacteria is rising, posing a global threat to clinical medicine and public health [6,7]. The problem is made worse by the expensive and long development time of new antibacterial drugs [8].

The appearance of new enzymes with new structures and the emergence of new mutations in known enzymes are the two main mechanisms driving the evolution of β-lactamases. TEM-1 was the first β-lactamase to be isolated from the blood of an infected patient in the early 1960s [9]. Mutations in this enzyme gave rise to the appearance of its variants that eventually made up the large TEM-type family of class A serine β-lactamases. There are currently 250 identified variants of TEM-1, differing from the parent enzyme by one to seven mutations. More than 60 structures of TEM-type β-lactamases in complex with inhibitors or antibiotic molecules at different resolutions are deposited to the Protein Data Bank, demonstrating the extensive research conducted on these enzymes. The high mutability of TEM-type β-lactamases made them an important model in biochemistry and enzymology, which facilitates the study of protein evolution and development of structure–function relationships [10,11,12].

In TEM-type β-lactamases, mutations were found in 30% of the residues [13]. Several β-lactamase mutations, referred to as “key”, have been identified; these mutations lead to an expansion of the substrate specificity in extended-spectrum β-lactamases (ESBLs) and a loss of sensitivity to mechanism-based inhibitors, like clavulanic acid and sulbactam [14,15]. These significant mutations account for only 10% of all β-lactamase mutations discovered. The analysis of the crystal structures showed that the key mutations lead to structural alterations, primarily affecting the configuration of the loops near the active site [16]. Mutant enzymes exhibit enhanced activity against cephalosporin antibiotics but worsen thermodynamic stability and kinetic activity toward their ancestral targets, penicillins.

Most of the mutating residues are located far from the active site on the peripheral part of the protein globule, and they are called “secondary”. Their role remains unclear; the exception is the M182T mutation found in different β-lactamases involving narrow-spectrum β-lactamases, ESBLs, and inhibitor-resistant mutant enzymes. It represents an example of a naturally occurring mutation that has evolved to alter the folding pathway of a protein and confer a selective advantage during the evolution of antibiotic resistance. It was discovered that M182T substitution exhibited a stabilizing effect, aiming at the compensation of enzymatic stability defects caused by the key mutations [17,18,19]. In β-lactamase TEM-1, the side chain of the M182 residue is located on the surface of the protein globule and is fixed by hydrophobic interactions with the acyl groups of R65, E63, and V159. The stabilizing role of the M182T mutation is explained by the formation of an additional hydrogen bond of the T182 residue with the amino group of the A185 residue, located on the boundary of the α-helix, as well as the formation of a hydrogen bond with the carbonyl group of the main chain of the E64 residue, which probably strengthens the connection between the α and α/β domains of the β-lactamases [20,21,22,23]. Due to this role, M182T is called a “global suppressor”, but the detailed molecular mechanism of this compensatory effect has not yet been established. Our previous analysis of the residue interaction networks (RINs) allowed for us to suggest the possible role of the R65 residue in stabilizing the protein globule, associated with a change in the mobility of the Ω-loop [22,24].

**Figure 1:**
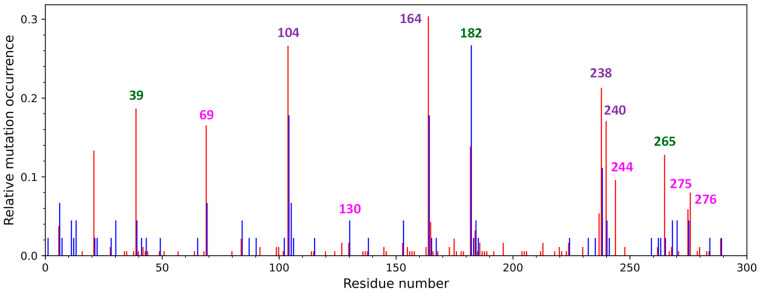
Relative occurrence of TEM-type β-lactamase mutations in 1990–2013 (red) and 2013–2024 (blue) years. The residue numbers responsible for expanded substrate specificity are violet and for resistance to inhibitors are pink; the most common secondary mutations are green.

The evolution of the appearance of key and secondary mutations that occurred in TEM-type β-lactamases is shown in Figure 1. Key mutations are more common than secondary ones, except for positions 182, 39, and 265. Remarkably, the percentage of enzymes with a mutation of the M182 residue has even doubled over the last decade, and currently the mutations of this residue are the most prevalent. This demonstrates how essential it is to study the mutations in this residue to understand how the protein globule of serine β-lactamases stabilizes and to find strategies for inactivating them.

Residue interaction networks (RINs) analysis of molecular dynamic trajectories revealed the influence of β-lactamase mutations on the stability of a protein globule and its constituent parts [24]. The RINs for M182T β-lactamase demonstrated redistribution of intra-protein contacts from residue M182 to residues of the Ω-loop. The latter is located at the entrance to the active site and comprises residues from R164 to D179, being an important structural element of serine β-lactamases. Presumably, the R65 residue plays a key role in changing the contact network of amino acid residues.

The aim of this study was to determine the influence of the amino acid residue at position 182 on the catalytic properties and thermal stability of β-lactamase TEM-1. For this, we performed saturating mutagenesis and isolated and purified TEM-1 M182X variants. For all TEM-1 variants, steady-state kinetic experiments were carried out with three substrates, penicillin, cephalothin, and ceftazidime; melting temperature and residual enzymatic activity after incubation at a temperature higher than the melting temperature were measured. To rationalize the experimental observations, we performed molecular dynamic simulations of TEM-1 variants, revealing the molecular basis of observed higher and lower thermoreactivation abilities compared with the wild-type enzyme.

## 2. Results

### 2.1. Periplasmic Expression of TEM Type β-Lactamase Genes in E. coli Cells

Saturation mutagenesis at position 182 in β-lactamase TEM-1 was carried out using the QuickChange method. Nineteen substitutions were introduced into the pET-bla expression vector, encoding all variants of amino acid substitutions, which are listed in Table S1. Then, *E. coli* BL21(*DE*3) cells were finally transformed with the expression vector coding for TEM-type β–lactamase variants with substitutions of the M182 residue. It is worth noting that, under the same conditions of cultivation, induction, isolation, and purification, the periplasmic fractions of all obtained β-lactamases were characterized by approximately the same level of expression of the recombinant protein. The exceptional variant was the enzyme with the M182P mutation characterized by a low level of synthesis. It can be assumed that the proline residue at position 182 affects the biosynthesis of this enzyme in the cell. All other eighteen variants of β-lactamase TEM-1 with M182X mutations were obtained in an active and soluble form.

### 2.2. Determination of Catalytic Parameters and Thermostability of Recombinant TEM-Type β-Lactamases with M182 Substitutions

A comparative study of the catalytic properties and thermal stability of the obtained homogeneous preparations of recombinant TEM-type β-lactamases allows for us to establish the individual effect of single amino acid substitutions of residue 182 on the properties of the enzymes. We determined the catalytic parameters of all recombinant β-lactamases in the reaction of hydrolysis of penicillin, cephalothin (1st generation cephalosporin), and ceftazidime (3rd generation cephalosporin). The data obtained showed that the mutants obtained do not differ in their properties from the wild-type enzyme TEM-1 (Table S2). The enzymes were most effective in the hydrolysis of penicillin, less effective in the hydrolysis of cephalothin, and practically ineffective in the hydrolysis of ceftazidime. This profile of substrate specificity corresponds to broad-spectrum β-lactamases (analogues of β-lactamase TEM-1). Thus, the replacement of the methionine residue at position 182 with other amino acids did not lead to a change in the catalytic properties of the enzymes compared to TEM-1.

Further, for all mutants together with the wild-type enzyme TEM-1, melting temperatures T_m_ were determined during thermal denaturation, in which protein structure disorders occur. For this purpose, differential scanning fluorimetry was used. The obtained data demonstrated that the resistance of the mutants to thermal denaturation differed (Table 1). To compare the changes in the properties of the enzymes with respect to the parent enzyme, TEM-1, the values of thermal shifts were calculated. All mutants are listed in Table 1 in the order of decreasing melting temperature. The M182T variant has the highest melting temperature (T_m_ = 56.2 °C), followed by M182S (T_m_ = 53.6 °C), M182A (T_m_ = 50.5 °C), and M182C (T_m_ = 50.6 °C). Four mutants, M182Q, M182N, M182H, and M182V, have the same melting temperature as TEM-1 (T_m_ = 48.9 °C). The remaining substitutions lead to a significant decrease in T_m_ compared to the wild-type enzyme; among them, the M182L variant has the lowest melting temperature (T_m_ = 41.1 °C).

The temperature of 60 °C was chosen to study the reactivation ability of mutant β-lactamases, as the melting temperatures, T_m_, for all considered enzymes are lower than this value. The measured T_m_ corresponds to the temperature at which half of the protein globules are present in a denatured state. The percentage of denatured globules at a given temperature (60 °C) increases with the decrease in the T_m_. The temperature of 60 °C was chosen because protein aggregation with subsequent precipitation was not observed, which was checked by the centrifugation of fractions. Thus, measurements of the residual enzymatic activity allow for the determination of whether the denaturation at the melting temperature is reversible or not.

**Figure 2:**
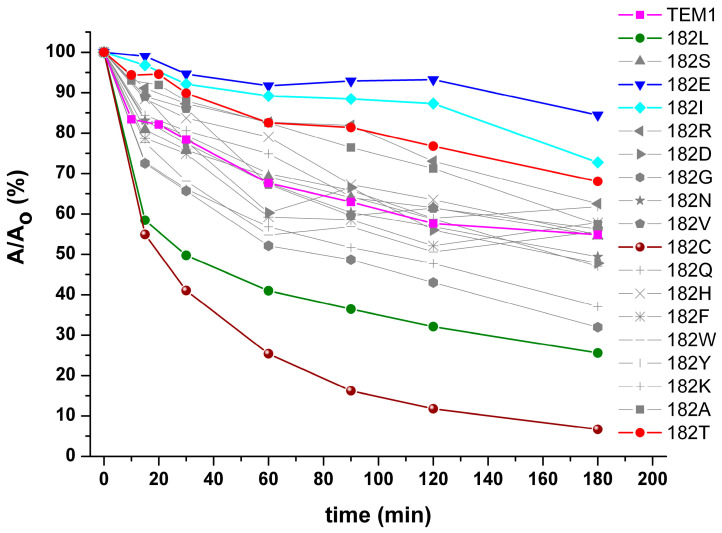
Time dependence of the relative residual activity of TEM-type β-lactamases with M182 residue substitutions. Enzyme concentration is 0.1 mg/mL, 50 mM sodium phosphate buffer pH 7.0. Incubation temperature is 60 °C.

The study of stability of the TEM-1 variants was carried out by incubating the enzymes at 60 °C for three hours and by periodic sampling of aliquots for determining catalytic activity during the denaturation process. These aliquots were first cooled to room temperature (25 °C) for 15 min, and then, the residual catalytic activity was measured. In control experiments, all samples were kept at room temperature, and activity was measured at the beginning and at the end of the experiment. The results are shown in Figure 2 and Table 1. Analysis of the inactivation curves suggests a multistage process of thermal inactivation, which correlates with previously reported data on the refolding of β-lactamase TEM-1 in the presence of guanidine hydrochloride [25]. Inactivation of beta-lactamase activity in control experiments was not observed for any of the enzyme variants.

Analysis of the obtained inactivation curves indicates that single substitutions of TEM-1 β-lactamase residue 182 result in multidirectional effects. Several mutant forms of β-lactamases have shown good reactivation ability compared to the wild-type enzyme TEM-1. Among them, the well-known natural variant with the M182T replacement stands out, as do M182E and M182I, which have higher reactivation abilities than the M182T substitution. An interesting fact is that the mutant M182I is characterized by a low melting temperature (T_m_ = 45.6 °C) compared to the other two mutants and, at the same time, is able to restore its activity well.

The data on reactivation allow for us to identify a second group of enzymes that are not able to effectively restore their activity after heating. These include M182C, M182L, M182G, and M182Q. The mutant M182C is characterized by the lowest reactivation ability, despite a sufficiently high melting temperature. Formation of intramolecular disulfide bonds in the M182C variant is unlikely due to steric limitations. The absence of dimerization of two protein molecules was shown by electrophoresis under non-reducing conditions.

### 2.3. Molecular Modeling of the Influence of Substitutions at M182 Residue

To rationalize the experimental observations, we performed molecular dynamic simulations of the wild-type enzyme; three variants with higher residual activity, M182E/I/T; three variants with lower residual activity, M182Q/L/G; and virtual alanine screening, E177A and R65A, to demonstrate the importance of interactions in the V159-R65-E177 triad.

**Figure 3:**
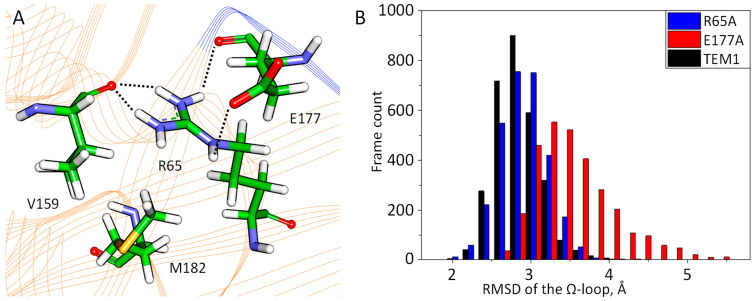
(**A**) Interactions of M182 and neighboring residues R65, E177, V159, and M182. Hydrogen bonds are shown in black dashed lines. The protein backbone is shown in cartoon representation and colored orange. The Ω-loop is blue. Here and on next Figures color code is the following: carbon—green, oxygen—red, nitrogen—blue, sulfur—yellow and hydrogen—white. (**B**) RMSD distributions for the Ω-loop for TEM-1 (black) and its R65A (blue) and E177A (red) variants.

According to the molecular dynamic simulations, the M182 amino acid residue influences the Ω-loop. The M182 residue forms hydrophobic interactions with V159 and with the γ-CH_2_ fragment of R65. These interactions stabilize a hydrogen bond between the carbonyl fragment of the V159 main chain and the R65 side chain. R65 also interacts with E177 of the Ω-loop (Figure 3A). The interactions of R65 and E177 affect the mobility of the Ω-loop, and their disturbance should lead to greater mobility of the part of the Ω-loop (residues 170–179). This was explicitly demonstrated in the virtual alanine screening of R65A or E177A in the molecular dynamic simulations (Figure 3B). An R65A mutation results in the appearance of two types of E177 conformations. It either forms salt bridges with R161 or is exposed to solvent. In the TEM-1 E177A variant, R65 does not interact with the carbonyl fragments of the main chain of V159 and A177. As a result, the part of the Ω-loop (170–179 residues) becomes more flexible and open.

Molecular dynamic trajectories were calculated for TEM-1 M182X (X = E, I, T, Q, G, L) variants to determine changes in the interaction of the V159-R65-E177 triad. Generally, for all considered systems, the 170–179 fragment of the Ω-loop is more flexible compared to the rest of the Ω-loop. It is even more flexible for variants with the disturbed interactions of E177 with other members in the triad.

**Figure 4:**
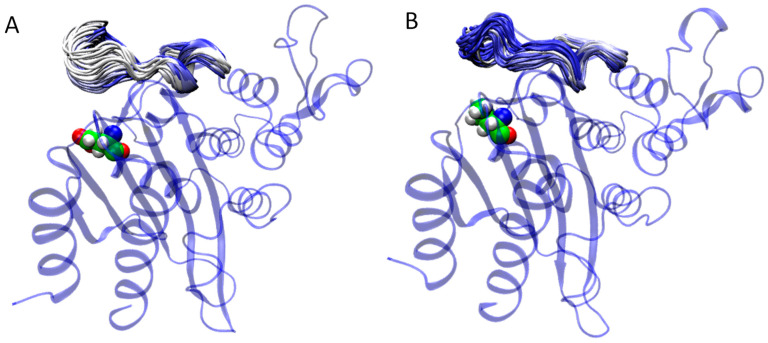
Alignment of MD trajectory frames for the TEM-1 M182E (**A**), with the mutation M182L (**B**). Several conformations of the Ω-loop along MD trajectories are shown to demonstrate the loop flexibility. The protein backbone is shown in cartoon representation and colored blue. The 182nd residue is shown in stick representation.

**Figure 5:**
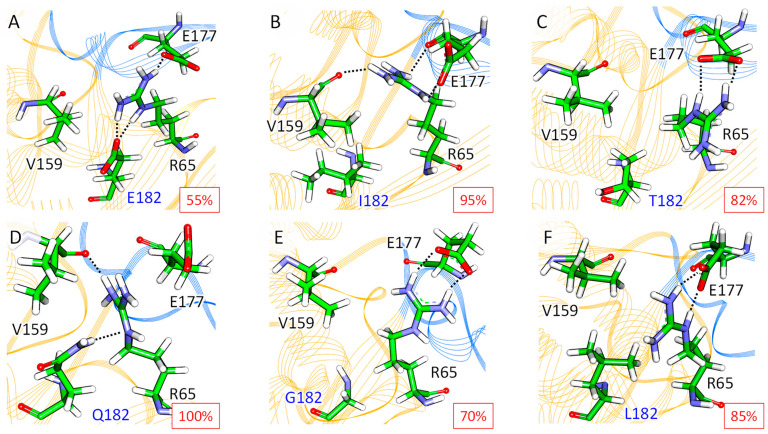
Interactions in the V159-R65-E177 region in the TEM-1 M182X variants. Upper panels correspond to the variants with higher residual activities, M182E (**A**), M182I (**B**), and M182T (**C**). Lower panels correspond to variants with lower residual activities, M182Q (**D**), M182G (**E**), and M182L (**F**). The protein backbone is shown in cartoon representation and colored orange, the Ω-loop is colored blue. Hydrogen bonds are shown by dotted lines. Representative structures and their corresponding occupancies are shown.

For β-lactamase TEM-1 M182E/I/T variants (Figure 4A) with higher residual activity, the flexibility of the 170–179 residues of the Ω-loop is higher than for systems with M182Q/L/G substitutions with lower residual activity (Figure 4B). The residual activity of TEM-1 with an M182E mutation was the highest. In this case, E182 joins the triad, forming salt bridges with R65, and the new E182-R65-E177 triad is formed (Figure 5A). Also, hydrophobic interactions between V159 and R65 via the hydrophobic fragment of E182 remain. M182I substitution does not affect interactions in the triad (Figure 5B). In the M182T variant, the γ-CH_3_ group of T182 forms hydrophobic interactions with the V159 side chain and γ-CH_2_ group of R65 (Figure 5C). The hydrogen bond between R65 and the backbone of V159 is not formed, but the R65-E177 salt bridge remains.

In the M182Q variant, Q182 competes with E177 for interactions with R65. As a result, Q182 forms a stable hydrogen bond with R65; the hydrogen bond between R65 and V159 is conserved, and E177 rarely interacts with R65 (Figure 5D). For the M182G variant, a chain of hydrophobic interactions, V159-M182-R65, found in the wild-type enzyme is disturbed (Figure 5E). R65 does not interact with V159, neither via hydrogen bond nor via hydrophobic interactions. The leucine residue at position 182 has a large hydrophobic side chain that leads to the conformational flexibility of R65. R65 interacts either with the V159 backbone or with the E177 carboxylate with the absence of the stable interactions within the V159-R65-E177 triad (Figure 5F). Thus, we demonstrate the importance of the V159-R65-E177 triad: For M182X variants with higher residual activity, the electrostatic and hydrophobic interactions remain or alter to compensating interactions, whereas for variants with reduced residual activity, some of these interactions are absent.

## 3. Discussion

The emergence and widespread distribution of antibiotic-resistant bacteria have prompted a thorough investigation of the enzymes responsible for different resistance mechanisms [26]. The investigation of β-lactamases, a large superfamily of serine and metallo-enzymes that confer resistance to β-lactam antibiotics, remains a subject of significant interest among them. The family of serine β-lactamases known as TEM-type, which includes the parent enzyme TEM-1 and over 200 of its mutant forms, provides a useful biological model for examining the impact of mutations on the stability and characteristics of enzymes in search of novel inhibitory strategies. The interest in studying mutations of this residue originated from the well-known compensatory effect of the M182T mutation on the stability of the protein globule; for this reason, it has been designated a “global suppressor” [17,18,19].

We studied all amino acid substitutions of the M182 residue in β-lactamase TEM-1 to determine their influence on the catalytic properties, the ability of enzymes for reversible thermal inactivation, and the melting temperature of the protein globule. For this purpose, all variants of beta-lactamase TEM-1 expression vectors with substitutions of the M182 residue were obtained. The recombinant β-lactamases were characterized by approximately the same level of expression; the exception being the M182P variant, which was characterized by a low level of synthesis, probably due to disturbances in the translation process.

The study of thermal inactivation of mutant forms of β-lactamases made it possible to identify the individual characteristics of the corresponding single amino acid substitutions on the ability of the enzymes to undergo reversible inactivation. It was shown that single substitutions on the surface regions of the protein globule of TEM-type β-lactamases can have multidirectional effects. A stabilizing effect is observed in the case of two mutants containing isoleucine and glutamic acid residues at position 182. The M182T mutation is the most studied of the secondary mutations in TEM type β-lactamases. The stabilizing effect on the protein globule was established for enzyme M182T variants in combination with key amino acid residue mutations [18]. It should be noted that all previously conducted experiments on the stability of various forms of β-lactamases describe the thermodynamic stability of enzymes, the quantitative expression of which is the melting temperature of the protein globule. The apparent contradiction can be explained within the framework of the theory of β-lactamase refolding [25,27,28]. In this work, the residual activity of the enzymes was measured after incubation at 60 °C, i.e., the ability of an enzyme to undergo reversible renaturation, which does not depend on the melting temperature of the protein globule. As was shown earlier, the refolding process of TEM-1 β-lactamase is complex and consists of at least five stages, which in turn consist of an initial fast phase (3–50 ms) and a final slow phase (300 or more ms) [27].

The stabilizing role of the M182T mutation is explained by the formation of an additional hydrogen bond of the T182 residue with the amino group of the A185 residue, located on the boundary of the α-helix, as well as the formation of a hydrogen bond with the carbonyl group of the main chain of the E64 residue, which probably strengthens the connection between the α and α/β domains of the β-lactamases [20,21].

Our previous analysis of the networks of intra-protein interactions in β-lactamases with the M182T substitution performed by RINs demonstrated the possible role of the R65 residue in stabilizing the protein globule, associated with a change in the mobility of the Ω-loop [22,24]. In β-lactamase TEM-1, the side chain M182 is located on the surface of the protein globule and is fixed by hydrophobic interactions with the acyl groups of R65 and V159. This leads to the fixation of the Ω-loop in a closed conformation, pressed against the protein globule, which increases its compactness. In this work, molecular dynamic simulations expanded a mechanistic explanation for this phenomenon. We revealed that the R65 residue is involved in the V159-R65-E177 triad, stabilized by both electrostatic and hydrophobic interactions. Amino acid substitutions that disturb this triad lead to a decrease in the thermal reactivation ability of the β-lactamase. The E177 residue is located on the Ω-loop; thus, new data confirm earlier assumptions about the impact of the conformation of this loop on the stabilization of the protein globule.

Saturation mutagenesis at position 182 performed in this work demonstrates pronounced tuning of thermoreactivation associated with amino acid substitutions. The important conclusion is that fine structural variations may considerably affect this property. For example, M182I and M182L, both having hydrophobic side chains of similar size, affect in opposite directions compared with the wild-type enzyme. The residual activity of these two variants differs by three-fold (Table 1). The introduction of a negatively charged M182D slightly influences the residual activity after 60 °C incubation compared with the wild-type TEM-1, whereas the TEM-1 M182E variant demonstrates the highest ability for reactivation among all considered enzymes. The M182T mutation in serine TEM-type β-lactamases continues to be common in pathogenic bacteria found in clinical settings. It is interesting that a β-lactamase variant with the replacement of M182I has recently been isolated. Our data indicate that it is also thermostable. The M182E variant has not yet been detected in clinical samples.

Our research highlights the value of using both molecular modeling and experimental saturation mutagenesis techniques to examine how secondary mutations affect β-lactamase characteristics. The investigation of the protein globule surface spots, which contain residues that impact a protein’s susceptibility to external stimuli, facilitates the discovery of novel targets for β-lactamase inhibition and a potential “hot spot”, such as position 182 in TEM-type β-lactamases, which govern a protein’s susceptibility to external stimuli, and facilitates the development of allosteric β-lactamase inhibition as a strategy for overcoming resistance.

## 4. Materials and Methods

### 4.1. Reagents

Reagents from Sigma-Aldrich (St. Louis, MO, USA; http://www.sigmaaldrich.com) and Difco (Franklin Lakes, LA, USA; http://www.bd.com) companies were used in this work. QIA Spin Miniprep Kit and QIAquick Gel extraction Kit (Tegelen, Netherlands; https://www.qiagen.com) were used to work with DNA samples. Restriction and DNA modification enzymes were produced by New England Biolabs (Ipswich, MA, USA; https://www.neb.com) and ThermoFisher Scientific (Waltham, MA, USA; https://www.thermofisher.com). The synthesis of oligonucleotides for sequencing and PCR was carried out at the Syntol company (Moscow, Russia; https://www.syntol.ru). DNA sequencing was carried out at Evrogen (Moscow, Russia; http://evrogen.ru). To determine protein concentration, a BCA test kit (Sigma-Aldrich) was used; SOURCE^TM^ 15Q chromatographic resin (Atlanta, GA, USA; http://www.gehealthcare.com) was used for protein ion-exchange purification. All aqueous solutions were prepared using Milli-Q deionized water (Millipore, Billerica, MA, USA; http://www.merckmillipore.com).

### 4.2. Microorganisms, Media, Plasmids, and Oligonucleotides

For genetic manipulations, *E. coli* DH5α strain and plasmid vector pET-24 were used for protein expression—*E. coli* BL21(*DE*3) (https://www.thermofisher.com). Cells were cultured in LB medium (0.5% yeast extract, 1% peptone, 0.5% NaCl) containing 50 mg/L kanamycin. To obtain competent cells, the culture was grown to OD_600_ 0.4–0.6 in 50 mL of LB medium; the cells were separated from the culture medium by centrifugation at 3500× *g* at 4 °C for 10 min. The cell pellet was resuspended on ice in TSS buffer (a buffer based on LB medium containing 10 g of PEG-6000, 5 mL of DMSO, and 0.6 g of MgCl_2_ per 100 mL; pH 6.5), kept for 1 h on ice, and frozen in liquid nitrogen and stored at −80 °C.

The search for nucleotide sequences of β-lactamase genes was carried out on the website of the Beta-Lactamase Data Base [23]. The alignment of β-lactamase sequences was carried out using the Multiple alignment program, Multalin DNA (http://npsa-pbil.ibcp.fr/, accessed on 12 July 2024). Translation of the DNA nucleotide sequence into an amino acid sequence was carried out on the ExPASy Translate tool server (https://web.expasy.org/translate/, accessed on 12 July 2024). Calculation of protein pI and Mw was performed on the same server (https://web.expasy.org/compute_pi/, accessed on 12 July 2024).

### 4.3. Introduction of Mutations into bla_TEM-1_ Using the Quick Change Method

The pET-24a(+) vector with the cloned TEM-1 β-lactamase gene (pET-*bla*) was used as a template [29]. 25-mer primers were used to introduce the mutations (Table S2). Amplification was carried out using *Pfu* polymerase in a total volume of 25 μL in 0.2 mL thin-walled tubes in a DNA amplifier Mastercycler gradient (Eppendorf, Hamburg, Germany) according to the following protocol: initial denaturation at 95 °C, 2 min; amplification, 15 cycles; denaturation at 95 °C, 30 s; primer annealing at 55 °C, 1 min; elongation at 72 °C, 7 min; final elongation at 72 °C, 10 min; cooling the mixture to +4 °C. Restriction enzyme *Dpn*I was added to the PCR mixture and incubated at 37 °C for 1 h, and then, it was used to transform *E. coli DH*5*α* cells.

### 4.4. Cultivation of E. coli Cells

*E. coli* BL21(DE3) cells were transformed with the pET-*bla* vectors and grown in LB medium with kanamycin (50 μg/mL). Cells were grown at 30 °C with stirring (180 rpm) until the optical absorption value at 600 nm was 0.8–1.2 units and then induced with 0.1 mM IPTG; cultivation was continued for 3 h. Cells were collected by centrifugation at 3000× *g* at 4 °C and stored at −20 °C.

### 4.5. Isolation and Purification of Recombinant Mutant Forms of TEM-Type β-Lactamases

To isolate the periplasmic fraction, the osmotic shock method was used. Cells were thawed on ice and resuspended in a buffer containing 20% sucrose, 1 mM EDTA, and 10 mM Tris/HCL, pH 8.0, and incubated on ice for 15 min. The resulting spheroplasts were removed by centrifugation at a speed of 10,000× *g* at 4 °C for 15 min. The supernatant was dialyzed against 10 mM Tris/HCL pH 8.0 and used for further purification. Anion exchange chromatography was performed on a SOURCETM 15Q column (10 cm × 0.75 cm^2^, GE HealthCare, Atlanta, GA, USA) equilibrated with the same buffer; the recombinant enzyme preparation was eluted in a linear gradient (0–300 mM NaCL, 2 mL/min). Enzymatic activity was determined in all fractions against a substrate CENTA (BD Biosciences, La Jolla, CA, USA) at 25 °C using a UV-1602 spectrophotometer (Shimadzu, Kyoto, Japan). The reaction mixture included 50 mM sodium phosphate buffer pH 7.0, substrate (50 μM), and an aliquot of the fraction. The formation of a chromogenic product was detected at a wavelength of 405 nm (∆ε_405_ = 6400 M^−1^ × cm^−1^) [30]. Fractions containing the active enzyme were stored at +4 °C or frozen at −20 °C. An additional purification step included gel filtration chromatography using a Superose^®^ 12 10/300 GL column (https://www.gelifesciences.com). The purity of the enzyme fractions was determined by SDS-PAGE. The protein concentration was determined spectrophotometrically. The extinction coefficient for beta-lactamase TEM-1 is ε_280_ = 28,085 M^−1^ cm^−1^; the values for the other mutants were calculated using the Protparam program (https://web.expasy.org/protparam/, accessed on 12 July 2024).

### 4.6. Determination of Catalytic Parameters of Recombinant ß-Lactamases

A solution of β-lactam antibiotics (penicillin, cephalothin, ceftazidime, each of 4 mM in 50 mM Na-phosphate buffer pH 7.0) and an enzyme aliquot (10 μL) were added to 50 mM sodium phosphate buffer pH 7.0. The concentration of β-lactamase in the assay was 12 nM. The total volume of the mixture was 1 mL. The reaction was initiated by adding the antibiotic solution at concentrations of 10, 20, 50, 100, and 200 µM to the solution containing the enzyme. The values of the initial rates of the enzymatic reaction were determined from the initial linear section of the kinetic curve of substrate consumption during the hydrolysis. The following values of extinction coefficient changes were used to calculate the decrease in substrate concentration: for penicillin, ∆ε_233_ = 1140 M^−1^ × cm^−1^; for cephalothin, ∆ε_262_ = 7660 M^−1^ × cm^−1^; for ceftazidime, ∆ε_260_ = 10,500 M^−1^ × cm^−1^ [31,32]. The values of the kinetic parameters (K_M_ and V_max_) were determined using a weighted Lineweaver–Burk linearization. The weights were taken as V_0_^4^/σ^2^(V_0_). In the calculations, it was assumed that the concentration of active centers of enzymes corresponds to the concentration of the enzyme in the reaction mixture.

### 4.7. The Study of the Thermal Inactivation of Recombinant ß-Lactamases

Thermal inactivation was carried out by incubation of the enzyme (0.1 mg/mL in 50 mM sodium phosphate buffer pH 7.0) at 60 °C for three hours with periodic sampling of aliquots after 10, 20, 30, 60, 90, 120, and 180 min. After cooling to room temperature, the residual activity of ß-lactamases was determined against a substrate CENTA at 25 °C. The reaction mixture included a substrate (50 μM) and an enzyme (12 nM) in 50 mM sodium phosphate buffer pH 7.0. The formation of a chromogenic product was detected at a wavelength of 405 nm (∆ε_405_ = 6400 M^−1^ × cm^−1^) [30]. The measurements were made in triplicate. To exclude possible sorption of the enzyme during incubation, protein concentrations were measured in all selected aliquots. Control experiments consisted of keeping the enzyme at room temperature and determining its activity. The enzyme activity at 25 °C was taken as 100% when determining the residual activity.

### 4.8. Thermal Shift Analysis of Recombinant ß-Lactamases

Melting temperatures (T_m_) were determined by differential scanning fluorimetry using Real Time PCR amplifier QuantStudio 5 (ThermoFisher Scientific, https://www.thermofisher.com). The reaction volume was 25 µL, containing 3 µM of recombinant ß-lactamase in 10 mM Tris-HCl with 100 mM NaCl pH 8.0 buffer and 1 µL 5xProteOrange (Lumiprobe RUS Ltd., Moscow, Russia; https://www.lumiprobe.com/). The thermal gradient was conducted between 25 °C and 95 °C with 1 °C/min intervals. Each thermal shift measurement was performed in triplicate. Fluorescence was read using the SyberGreen filter channel of the QuantStudio 5, and the T_m_ values were determined by plotting the first derivative of the fluorescence emission as a function of temperature.

### 4.9. Molecular Modeling

The TEM-1 molecular model was obtained from the crystal structure PDB ID: 1M40 [33] with a resolution of 0.85 Å. The TEM-1 full-atom model was solvated in a rectangular water box with the distance from any atom of a protein to the cell boundaries exceeding 12 Å. Then, chlorine anions were added to the system to neutralize the total charge of the cell. After that, in silico alanine screening of R65 and E177 residues was carried out for the subsequent study of the influence of the interaction between R65 and E177 on the mobility of the Ω-loop. The set of M182X variants were constructed by replacement of M182 by threonine, glutamate, isoleucine, leucine, glutamine, and glycine residues. All amino acid substitutions and system preparation, including solvation and neutralization, were carried out in the VMD program [34]. Classical molecular dynamics (MD) simulations were performed using the CHARMM36 [35,36] force field for the protein and TIP3P [37] for water molecules. The parameters of the MD simulations were T = 300 K, p = 1 atm, and 1 fs integration time step. The length of the trajectory MD was 500 ns for the study of the influence of the interaction between R65 and E177 on the mobility of the Ω-loop and 100 ns for the analysis of the M182X mutants. Calculations were performed using the NAMD program package [38]. RMSD was calculated over trajectory for all model systems (Figure S1).

## 5. Conclusions

In this study, a complete set of recombinant TEM-1 β-lactamases with all possible substitutions of methionine 182 for other amino acid residues except proline was investigated. Given that M182T has remained constant throughout the evolution of β-lactamases and is frequently found in clinical strains, there is a great interest in understanding the mechanism of stabilizing the enzyme’s protein globule through replacement. Experimental studies demonstrate that any variants of substitutions of residue 182 do not change substrate specificity or the catalytic activity of the enzymes. At the same time, all variants of β-lactamases with the replacement of this residue differ significantly both in thermodynamic stability and in their ability for refolding to the catalytically active form after thermal denaturation at elevated temperature. Molecular dynamic simulations of TEM-1 variants revealed the role of the V159-R65-E177 triad as a crucial structural component. Enzymes that possess specific M182 substitutions, which cause a structural disruption in this triad, exhibit a decreased capacity for reactivation.

## Figures and Tables

**Table 1:** Melting temperatures (T_m_) and residual activities for mutants of β-lactamase TEM-1 with substitutions at position 182. ∆T is the melting temperature change relative to T_m_ for TEM-1 (∆T = T_m_ − T_m_ (TEM-1)). Relative residual enzymatic activity towards substrate CENTA was determined after 3 h of incubation at 60 °C (shown in last column).

M182 Substitution in β-Lactamase TEM-1	T_m_, °C	∆T, °C	Relative Residual Activity, %
M182 (wild-type)	48.9 ± 0.2	0	55
M182T	56.2 ± 0.2	7.3	68
M182S	53.6 ± 0.2	4.7	55
M182A	50.5 ± 0.1	1.6	57
M182C	50.6 ± 0.1	1.7	7
M182Q	49.1 ± 0.1	0.2	47
M182N	49.1 ± 0.2	0.2	49
M182H	48.6 ± 0.1	−0.3	55
M182V	48.1 ± 0.2	−0.8	56
M182E	47.0 ± 0.1	−1.9	84
M182K	46.6 ± 0.1	−2.3	37
M182W	46.2 ± 0.1	−2.7	56
M182Y	45.7 ± 0.2	−3.2	62
M182I	45.6 ± 0.1	−3.3	73
M182G	45.0 ± 0.1	−3.9	32
M182R	44.2 ± 0.1	−4.7	63
M182F	44.1 ± 0.1	−4.8	58
M182D	42.3 ± 0.1	−6.6	48
M182L	41.1 ± 0.1	−7.8	26

## Data Availability

The original contributions presented in this study are included in the article/Supplementary Materials; further inquiries can be directed to the corresponding author.

## Supplementary Materials

The following supporting information can be downloaded at: https://www.mdpi.com/article/10.3390/ijms25147691/s1.
