# Supplementary material for: Saturation Mutagenesis and Molecular Modeling: The Impact of Methionine 182 Substitutions on the Stability of β-Lactamase TEM-1
Source: Int J Mol Sci. 2024 Jul 13;25(14):7691. doi: 10.3390/ijms25147691 (PMC11276661; doi:10.3390/ijms25147691)
Supplement: Supplementary file 1 [file ijms-25-07691-s001.zip › ijms-3090072-supplementary.pdf]

Table S1. Sequences of primers for introducing M182 substitutions in TEM-type recombinant β-lactamases.

M182 substitution	The encoding triplet introduced	Primer direction	Sequence (5'-3')
M182	ATG	Forward	GTGACACCACGATGCCTGCAGCAATG
		Reverse	CATTGCTGCAGGCATCGTGGTGTCAC
M182T	ACG	Forward	GTGACACCACGACGCCTGCAGCAAT
		Reverse	CATTGCTGCAGGCGTCGTGGTGTCAC
M182I	ATT	Forward	GTGACACCACGATTCCTGCAGCAATG
		Reverse	CATTGCTGCAGGAATCGTGGTGTCAC
M182N	AAT	Forward	GTGACACCACGAATCCTGCAGCAATG
		Reverse	CATTGCTGCAGGATTCGTGGTGTCAC
M182K	AAG	Forward	GTGACACCACGAAGCCTGCAGCAATG
		Reverse	CATTGCTGCAGGCTTCGTGGTGTCAC
M182S	AGC	Forward	GTGACACCACGAGCCCTGCAGCAATG
		Reverse	CATTGCTGCAGGGCTCGTGGTGTCAC
M182R	AGG	Forward	GTGACACCACGAGGCCTGCAGCAATG
		Reverse	CATTGCTGCAGGCCTCGTGGTGTCAC
M182V	GTG	Forward	GTGACACCACGGTGCCTGCAGCAATG
		Reverse	CATTGCTGCAGGCGCCGTGGTGTCAC
M182A	GCG	Forward	GTGACACCACGGCGCCTGCAGCAATG
		Reverse	CATTGCTGCAGGCGCCGTGGTGTCAC
M182D	GAT	Forward	GTGACACCACGGATCCTGCAGCAATG
		Reverse	CATTGCTGCAGGATCCGTGGTGTCAC
M182E	GAG	Forward	GTGACACCACGGAGCCTGCAGCAATG
		Reverse	CATTGCTGCAGGCTCCGTGGTGTCAC

M182G	GGG	Forward	GTGACACCACGGGGCCTGCAGCAATG
		Reverse	CATTGCTGCAGGCCCCGTGGTGTCAC
M182F	TTT	Forward	GTGACACCACGTTTCCTGCAGCAATG
		Reverse	CATTGCTGCAGGAAACGTGGTGTCAC
M182L	TTG	Forward	GTGACACCACGTTGCCTGCAGCAATG
		Reverse	CATTGCTGCAGGCAACGTGGTGTCAC
M182Y	TAC	Forward	GTGACACCACGTACCCTGCAGCAATG
		Reverse	CATTGCTGCAGGGTACGTGGTGTCAC
M182C	TGC	Forward	GTGACACCACGTGCCCTGCAGCAATG
		Reverse	CATTGCTGCAGGGCACGTGGTGTCAC
M182W	TGG	Forward	GTGACACCACGTGGCCTGCAGCAATG
		Reverse	CATTGCTGCAGGCCACGTGGTGTCAC
M182P	CCG	Forward	GTGACACCACGCCGCCTGCAGCAATG
		Reverse	CATTGCTGCAGGCGGCGTGGTGTCAC
M182H	CAC	Forward	GTGACACCACGCACCCTGCAGCAATG
		Reverse	CATTGCTGCAGGGTGCGTGGTGTCAC
M182Q	CAG	Forward	GTGACACCACGCAGCCTGCAGCAATG
		Reverse	CATTGCTGCAGGCTGCGTGGTGTCAC

Table S2. The values of the catalytic parameters of recombinant TEM-type β-lactamases with substitutions of residue 182 in the hydrolysis of penicillin, cephalotin, and ceftazidime.

M182 substitu-tion	Penicillin		Cephalotin		Ceftazidime	
	K_M, μM	*Vmax/[E]$_0$, 1/s*	*K_M, μM*	*Vmax/[E]$_0$, 1/s*	*K_M, μM*	*Vmax/[E]$_0$, 1/s*
M182	75±8	1280±150	310±35	150±20	900±100	0.02±0.01
M182L	71±8	1140±150	300±35	145±20	970±110	0.02±0.01
M182S	73±8	1110±150	325±35	130±20	990±100	0.02±0.01
M182E	80±8	1070±150	350±35	110±20	935±110	0.02±0.01
M182I	78±8	1100±150	330±35	125±20	980±100	0.02±0.01
M182R	69±8	1130±150	340±35	130±20	970±110	0.02±0.01
M182D	72±8	1090±150	300±35	142±20	990±100	0.02±0.01
M182G	75±8	1170±150	335±35	145±20	980±110	0.02±0.01
M182N	70±8	1150±150	345±35	135±20	990±100	0.02±0.01
M182V	77±8	1120±150	340±35	140±20	960±110	0.02±0.01
M182C	75±8	1180±150	330±35	130±20	990±100	0.02±0.01
M182Q	80±8	1170±150	350±35	145±20	970±110	0.02±0.01
M182H	72±8	1160±150	335±35	145±20	1000±100	0.02±0.01
M182F	77±8	1140±150	355±35	135±20	980±110	0.02±0.01
M182W	74±8	1170±150	340±35	130±20	990±100	0.02±0.01
M182Y	78±8	1160±150	350±35	145±20	990±110	0.02±0.01
M182K	75±8	1180±150	335±35	145±20	970±100	0.02±0.01
M182A	80±8	1150±150	350±35	140±20	980±110	0.02±0.01
M182T	72±8	1200±150	330±35	150±20	990±100	0.02±0.01

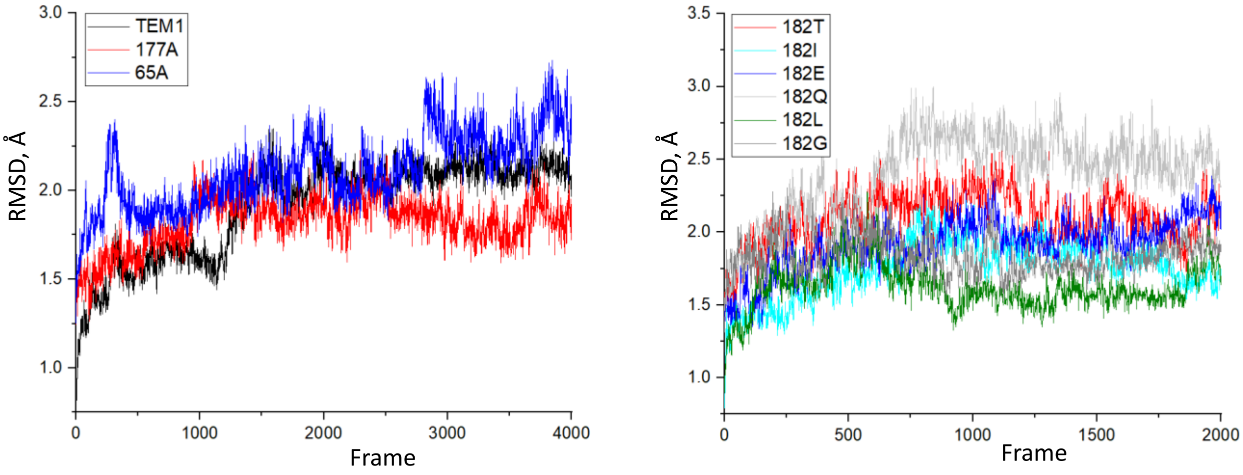

Figure S1. RMSD calculated along MD trajectories for all considered systems. The alignment is made over the heavy atoms of the initial structure.
